# Cases from Practice

**Published:** 1895-02

**Authors:** Lawrence M. Stanton

**Affiliations:** New York


### CASES FROM PRACTICE.

LAWRENCE M. STANTON, M. D., NEW YORK.

I select the following cases to report, as some of them emphasize symptoms in the provings which are not over-familiar, while others, perhaps, will show familiar symptoms in some new aspect or combination. All of them are examples of the efficacy of the high potency in single dose.

The potencies used are Dr. Fincke's.

### I. AORTIC INSUFFICIENCY.

A lady sixty-six years of age has for many years suffered from heart trouble. On auscultation this proved to be a very pronounced case of aortic insufficiency.

She had recently had an attack of bronchitis, and this together with tincture of Digitalis, prescribed by her physician, brought on the condition in which I found her.

Heart very feeble, irregular, intermittent, rapid—now fast, now slow.

Respiration rapid, shallow, with decided dyspnœa. The heart and respiration very much worse lying down. At night she had to be propped up in bed and slept only in snatches. She could not take a few steps in her room without very much increasing the heart's action and becoming faint.

Throbbing here and there throughout the body, especially marked in carotids, where it was painful, and in left hypochondrium. The heart was worse during the night and especially toward morning.

She was often troubled, on falling off to sleep, by the sensation of saliva trickling down her throat, and this apparently was the case.

Prostration was extreme and it seemed death was not far off.

Kali-carb.$^{19m}$, one dose.

Improvement began at once, the symptoms rapidly disappeared.

She soon slept as she had not for weeks, sometimes without once waking, and there was no longer the need of being propped up.

She walked about her room without palpitation or discomfort, and soon was able to get out of the house.

The remedy had to be repeated once or twice subsequently, in the same potency.

With such a valvular lesion the patient is not a well woman, but this was a year ago, and I recently heard that she was living in a smaller city and doing well, going out and about.

The symptom "has only been able to sleep sitting up, otherwise saliva would run down the throat," will be found as quoted under Heart, in Hering's *Guiding Symptoms.*

I have not been able to find that throbbing of carotids or in neck was distinctly characteristic of Kali-carb. But pulsations and ebullitions are common enough under this remedy, and Hering gives "painful throbbing in clavicle."

### II. Coryza.

The patient, a woman.

Nose very much stopped; severe headache in forehead, which was a bruised feeling, and was very much worse on motion. Tickling in pharynx, causing an almost constant cough.

Rumex 10m, one dose.

The headache—this bruised feeling—began to disappear in a couple of hours. Violent running from the nose and sneezing appeared, which, together with the tickling cough, soon vanished.

Lee, in his Repertory, gives four remedies that have bruised feeling in the head, worse on motion: Caps., China, Rumex, and Tellur. Hepar has bruised pain in the forehead, worse on moving the eyes.

In this case the constant tickling cough, so like Rumex, came from the pharynx, instead of from the usual situation—the supra-sternal fossa.

### III. Coryza.

For the past six weeks this patient, a woman, has had the following symptoms: Every morning, beginning when getting up, there has been profuse running from the nose, clear discharge, like water, with much sneezing.

This continued until 10 o'clock, when the running and sneezing would entirely cease, and the nose become very much obstructed, and remained so for the rest of the day. Each day was the same; at 10 o'clock the same change of symptoms.

℞. Nat-mur. 10m.

The following day there was decided improvement; then the day after an aggravation, which lasted twenty-four hours, when the whole condition disappeared.

The remedy in this case produced an amelioration at once; then a day of aggravation, to be succeeded by the cure.

### IV. Flatulent Dyspepsia.

A child, six years old, has suffered for the past two years from flatulent indigestion.

Abdomen enormously distended, rumbling in the abdomen, and almost constant passing of large quantities of wind. This was so incessant that the parents were ashamed to go anywhere with the child or have any one come to their home.

The boy suffered so from this drum-like distention that he would lie, often for the greater part of the day, upon his belly on the floor or bed.

The bowels were irregular. Occasionally he would vomit.

Carbo-veg. was, of course, the remedy, and one dose of the 4M potency cured speedily.

There has been so far not the least return of his symptoms, now six months since the remedy was given.

This case had been under allopathic treatment two years without beneficial result, and a specialist, one whose name is known throughout the land, could advise nothing better than to introduce the stomach-tube and wash out the stomach. The advice was not followed.

### V. Cornu-Cutaneum.

A case that is interesting, principally on account of the curative action of the homœopathic remedy, but also owing to the rarity of such horns in the human being. In the lower animals they are sufficiently common.

This horn occurred in a lady of about fifty-five years of age, and was situated on the end of one of her fingers. The growth measured nearly half an inch in length; in form it was conical; in consistency hard and dry. Every now and then there would be considerable inflammation about its base, with shooting pains here, and running up the hand. The inflammation often resulted in suppuration, and several times the finger had been lanced.

There was much pain and inflammation when I first saw the finger, and the lady feared the growth would have to be removed with the knife.

Silicea$^{cm}$, one dose.

In two weeks the horn had entirely disappearad.

This was three years ago, and there was no reproduction.

### VI. Housemaid's Knee.

This occurred in a man, an expressman by occupation.

It had resulted from the habit of bearing the weight of a trunk or box upon the patella of his right knee, and lifting the load thus into his wagon.

There was no swelling of the knee-joint proper. The swelling was between the integument and patella, and the bursa in this situation was undoubtedly the seat of trouble.

The swelling was marked, and looked like a large pad upon the knee. It extended far beyond the upper and lower borders of the knee-pan. The only subjective symptoms were a feeling of distention and weakness of the knee.

The village doctor said he would have to tap the swelling and draw off the fluid. Arnica was given, but with no effect.

There were few subjective symptoms upon which to prescribe, and thinking of the action of Apis on serous and synovial membranes, I gave one dose of this remedy in the 5 M potency. There was a sharp aggravation followed by a speedy disappearance of the dropsy.

The knee still remains sound, and it is more than a year since it was cured.

